# Supplementary material for: D,L-Lysine-Acetylsalicylate + Glycine (LASAG) Reduces SARS-CoV-2 Replication and Shows an Additive Effect with Remdesivir
Source: Int J Mol Sci. 2022 Jun 21;23(13):6880. doi: 10.3390/ijms23136880 (PMC9266999; doi:10.3390/ijms23136880)
Supplement: Supplementary file 1 [file ijms-23-06880-s001.zip › ijms-1787708-supplementary.pdf]

International Journal of
Molecular Sciences

MDPI

Article

D,L-lysine-acetylsalicylate + glycine (LASAG) reduces SARS-CoV-2 replication and shows an additive effect with remdesivir

Johannes Jungwirth [1], Clio Häring [1], Sarah König [1], Liane Giebeler [1], Heena Doshi [2], Christian Brandt [3], Stefanie Deinhardt-Emmer [4], Bettina Löffler [4] and Christina Ehrhardt [1,*]

[1] Section of Experimental Virology, Institute of Medical Microbiology, Center for Molecular Biomedicine (CMB), Jena University Hospital, Hans-Knoell-Str. 2, D-07745 Jena, Germany; johannes.jungwirth@med.uni-jena.de (J.J.); clio.haering@med.uni-jena.de (C.H.); sarah.koenig2@med.uni-jena.de (S.K.); liane.giebeler@med.uni-jena.de (L.G.); christina.ehrhardt@med.uni-jena.de (C.E.)
[2] Institute of Molecular Cell Biology, Center for Molecular Biomedicine (CMB), Jena University Hospital, Hans-Knoell-Str. 2, D-07745 Jena, Germany; heena.doshi@med.uni-jena.de (H.D.)
[3] Institute for Infectious Diseases and Infection Control; Jena University Hospital, Am Klinikum 1, D-07747 Jena, Germany; christian.brandt@med.uni-jena.de (C.B.)
[4] Institute of Medical Microbiology; Jena University Hospital, Am Klinikum 1, D-07747 Jena, Germany; stefanie.deinhardt-emmer@med.uni-jena.de (S.D.-E.); bettina.loeffler@med.uni-jena.de (B.L.)
* Correspondence: christina.ehrhardt@med.uni-jena.de; Tel.: 0049-(0)3641-9395700

Supplementary Materials:

The following supporting information can be downloaded at: www.mdpi.com/xxx/s1,

Figure S1: Structural formula of D,L-lysine acetylsalicylate + glycine.

Figure S2: LASAG reduces SARS-CoV-2 infection and replication, pro-inflammatory cell signaling and cytokine and chemokine synthesis *in vitro.*

Figure S3: LASAG treatment affects the endothelial cell morphology in the chip model, but not in mono cell culture assays.

Figure S4: LASAG and remdesivir show additive inhibitory effects on SARS-CoV-2 replication.

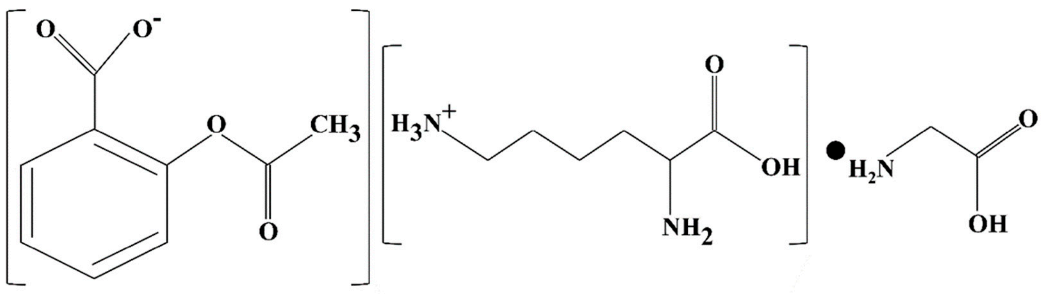

Supplementary Figure S1. Schematic representation of the structural formula of D,L-lysine-acetylsalicylate + glycine according to [34,35]. The two amino acids lysine and glycine improve the stability and tolerability of the active compound ASA.

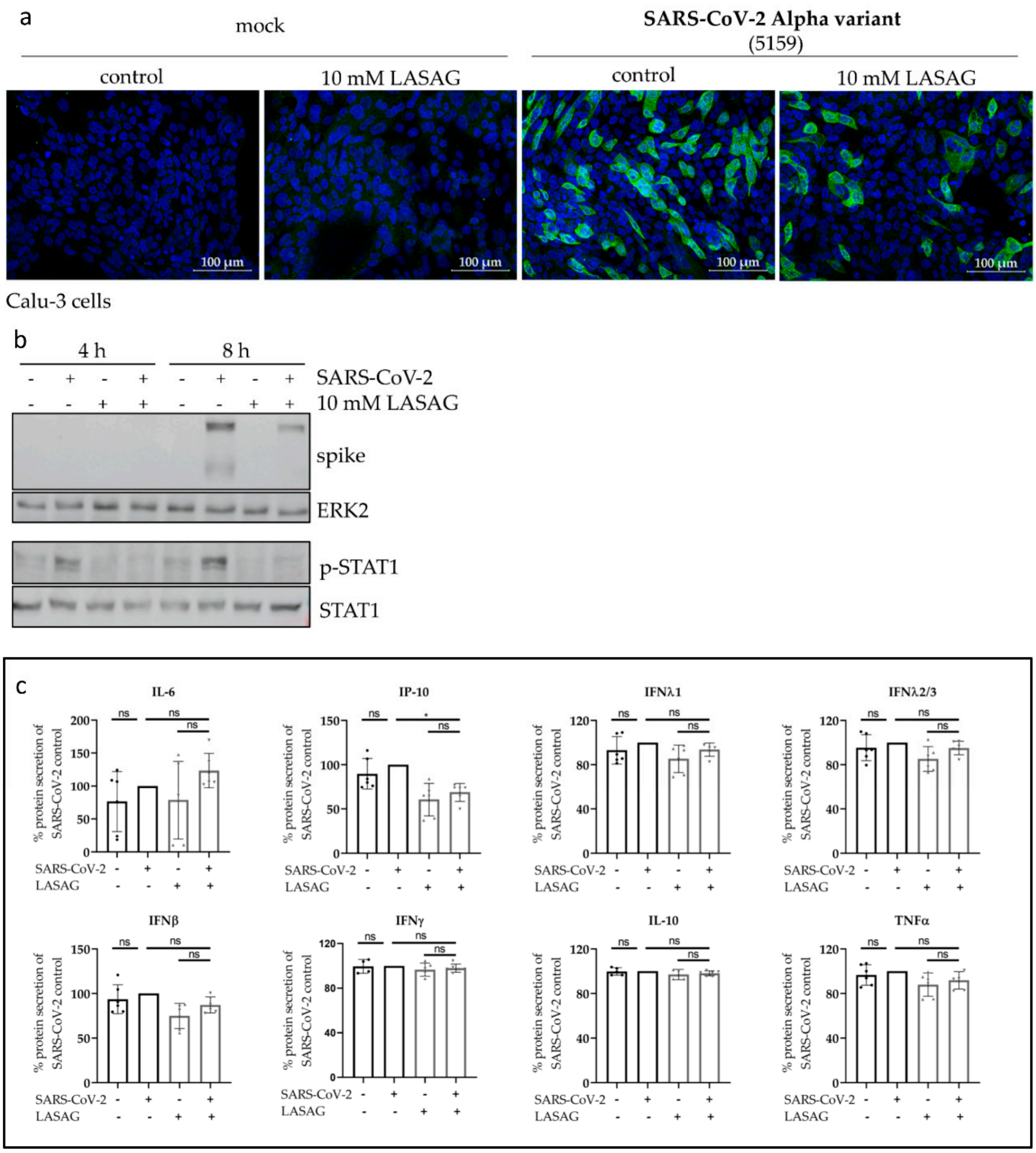

Supplementary Figure S2. LASAG reduces SARS-CoV-2 infection and replication, pro-inflammatory cell signaling and cytokine and chemokine synthesis in vitro. Calu-3 cells were left untreated or were incubated with 10 mM LASAG for 1 h. Subsequently, cells were left uninfected or were infected with the SARS-CoV-2 Alpha variant (isolate 5159) at 5 MOI in absence and presence of LASAG for 2 h. After a medium change cells were further incubated in absence and presence of LASAG. (**a**) In immunofluorescence microscopy SARS-CoV-2 spike protein was detected at 8 h p.i. by a spike-specific antibody and an Alexa Fluor 488-conjugated goat anti-mouse IgG (green). The nuclei were stained with Hoechst 33342 (blue). Immunofluorescence microscopy was acquired by use of the Axio Observer.Z1 instrument (Zeiss) with a x200 magnification. Scale bars represent 50 µm. Shown are representative example pictures of three independent experiments. (**b**) At 4 h p.i. and 8 h p.i. total cell lysates were used to investigate the expression of the viral spike protein and phosphorylated STAT1 (pY701) by Western blot analysis. ERK2 and STAT1 served as the loading controls. Shown are representative example blots of three independent experiments. (**c**) Supernatants were collected 8 h p.i. and different factors were analysed by flow cytometry (LEGENDplex™) using an anti-virus response panel. Means (± SD) of three independent experiments including two biological replicates are shown. The mean levels of untreated, infected samples were arbitrarily set to 100%. (**a-c**) After normalization, one way ANOVA followed by Dunnett`s multiple comparison test was peformed (ns, non-significant; *, P<0.0332; **, P<0.0021; ***, P<0.0002; ****, P<0.0001).

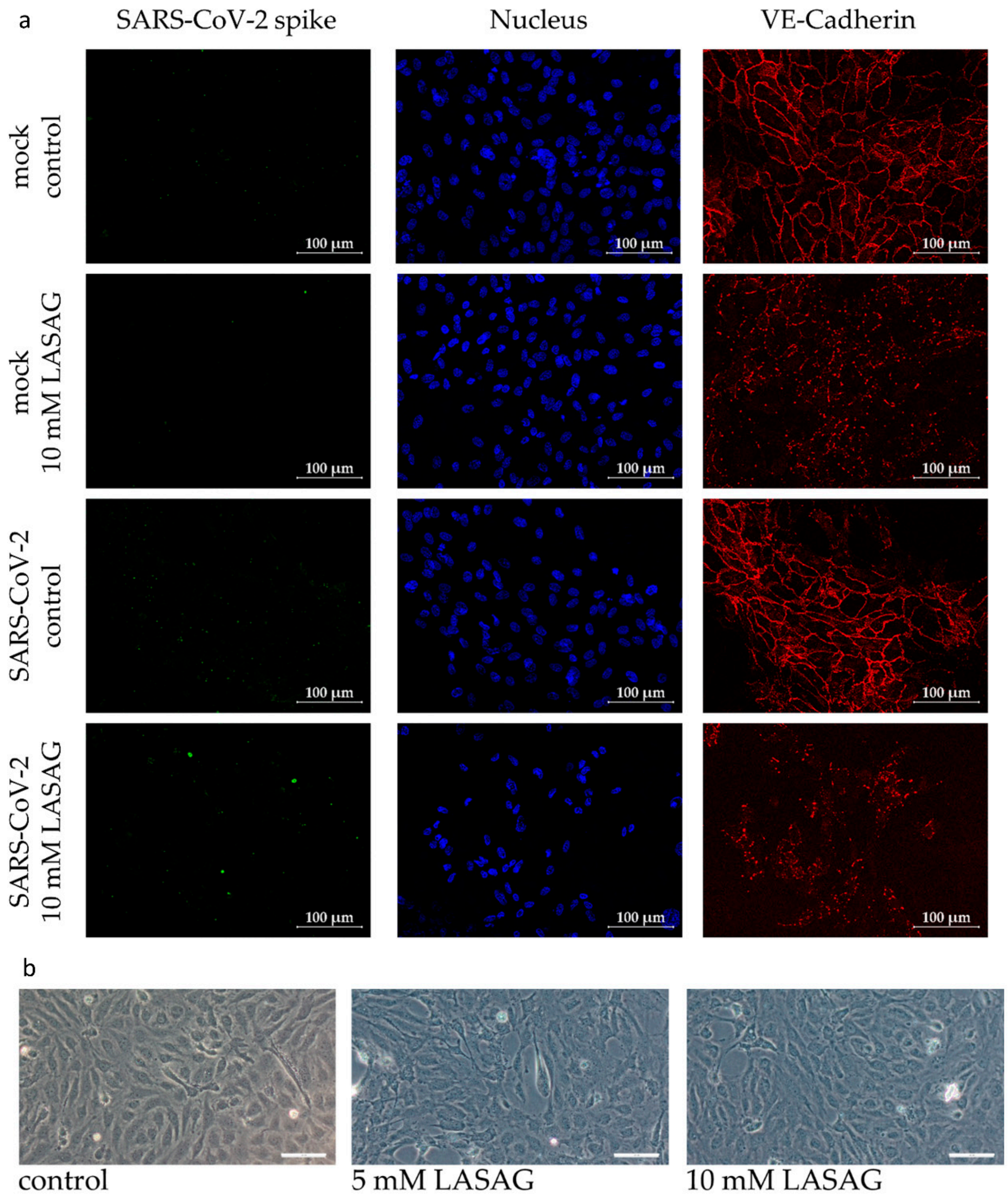

Supplementary Figure S3. LASAG treatment affects the endothelial cell morphology in the chip model, but not in mono cell culture assays. (**a**) Both, the epithelial and endothelial chamber were left untreated or were incubated with 10 mM of LASAG for 1 h. Subsequently, the chambers were left uninfected or were infected with the SARS-CoV-2 Alpha variant (isolate 5159) at 1 MOI in absence and presence of LASAG for 3 h. After a medium change cells were further incubated in absence and presence of LASAG. At 28 h p.i. immunofluorescence staining of the endothelial layer was performed and the SARS-CoV-2 spike protein was visualized via a spike-specific antibody and an Alexa Fluor 488-conjugated goat-anti mouse IgG antibody (green). The nuclei were stained with Hoechst 33342 (blue). The VE-Cadherin of the endothelial cells was visualized by an anti-VE-Cadherin-specific antibody and a Cy5-conjugated goat-anti rabbit IgG antibody (red). Scale bars represent 100 µm. (**b**) HUVECs cultured in 12-well plates were left untreated or were incubated with 10 mM LASAG for 24 h. Microscopy images were taken with an Axio Vert.A1 (Zeiss). Scale bars represent 50 µm.

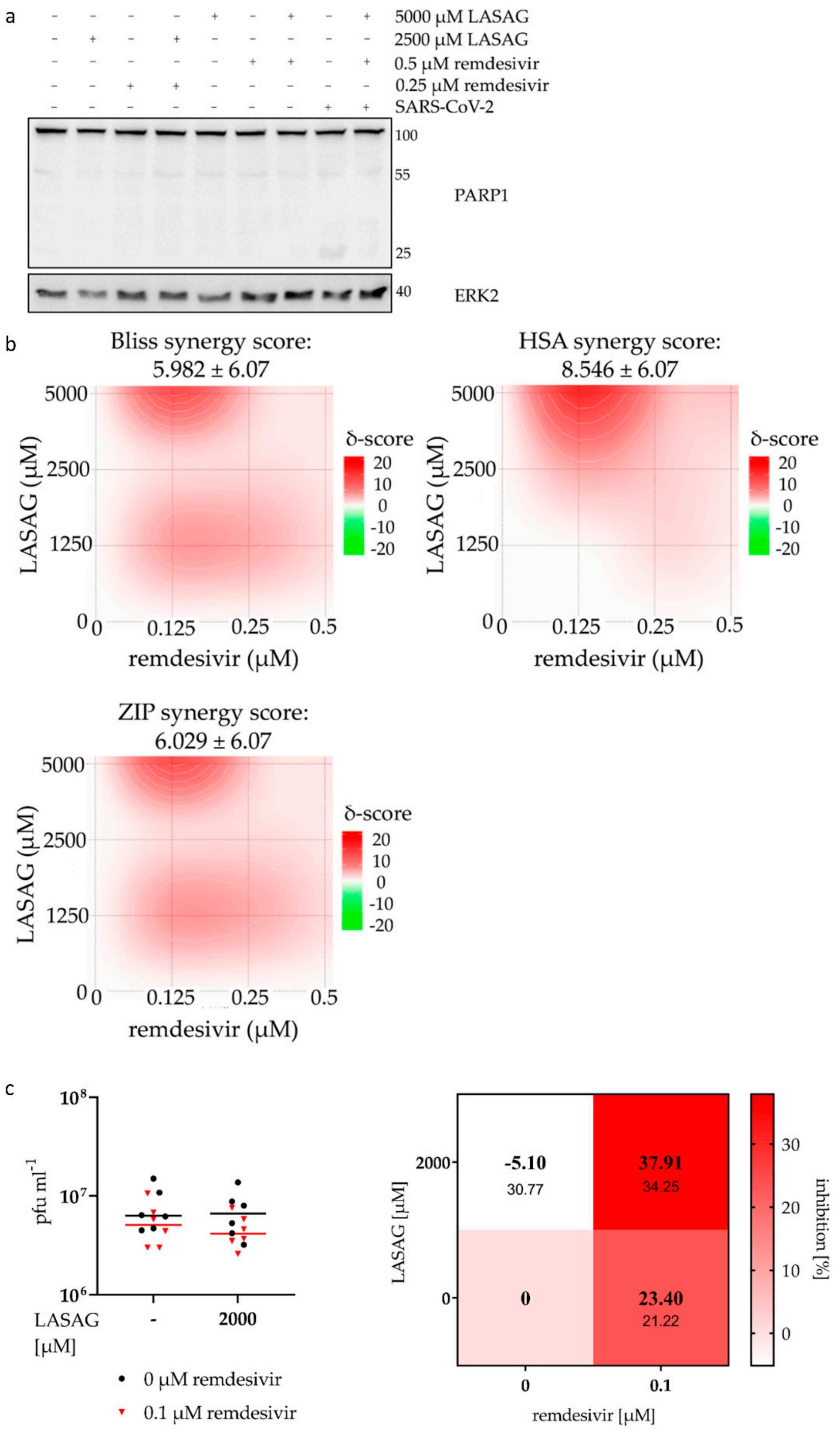

Supplementary Figure S4. LASAG and remdesivir show additive inhibitory effects on SARS-CoV-2 replication**.** Calu-3 cells were left untreated or were incubated with the LASAG-remdesivir combinations for 1 h. Subsequently, cells were infected with the SARS-CoV-2 Alpha variant (isolate 5159) at 0.5 MOI in absence and presence of the LASAG-remdesivir combinations for 2 h. After a medium change cells were further incubated in absence and presence of the LASAG-remdesivir combinations. (**a**) Lysates of experiments performed with the highest concentrations of LASAG and remdesivir

were used in Western Blot analysis to determine PARP1 cleavage. ERK2 served as loading control. (**b**) Bliss independence, highest single agent (HSA) and zero interaction potency (ZIP) reference models were used to assess the interaction landscapes and to identify areas of synergy. Interaction surfaces are colour coded according to the synergy scores of the responses [45]. (**c**) Even low concentrations of LASAG and remdesivir show an additive inhibitory effect on SARS-CoV-2-replication. In the left panel means of pfu ml^{-1} of three independent experiments including two biological samples and in the right panel the calculated inhibition (and SD) were used to evaluate the interactions of LASAG and remdesivir and are depicted in a heatmap.

References

34. Müller, C.; Karl, N.; Ziebuhr, J.; Pleschka, S. D, L-lysine acetylsalicylate + glycine Impairs Coronavirus Replication. Journal of Antivirals & Antiretrovirals 2016, 08, doi:10.4172/jaa.1000151.

35. Rübsamen-Schaeff, H.; Buschmann, H.; Mannhold, R.; Holenz, J. New Drug Development for Known and Emerging Viruses; Wiley: 2022.

45. Ianevski, A.; Giri, A.K.; Aittokallio, T. SynergyFinder 2.0: visual analytics of multi-drug combination synergies. Nucleic Acids Res 2020, 48, W488-W493, doi:10.1093/nar/gkaa216.

All here listed references are also listed with the same numbers within the manuscript.
